# Salivary Flow Rate During Toothbrushing

**DOI:** 10.3290/j.ohpd.b3601691

**Published:** 2022-11-23

**Authors:** Anina M. Pulfer, Thomas Attin, Florian J. Wegehaupt

**Affiliations:** a Dentist, Clinic of Conservative and Preventive Dentistry, Center for Dental Medicine, University of Zürich, Zürich, Switzerland. Performed the experiments in partial fulfilment of requirements for a Doctor’s degree and wrote the manuscript.; b Professor and Director, Clinic of Conservative and Preventive Dentistry, Center for Dental Medicine, University of Zürich, Zürich, Switzerland. Contributed substantially to the discussion and proofread the manuscript.; c Head of Division of Preventive Dentistry and Oral Epidemiology, Clinic of Conservative and Preventive Dentistry, Center for Dental Medicine, University of Zürich, Zürich, Switzerland. Research idea, hypothesis, experimental design, contributed substantially to discussion and writing the paper, proofread the manuscript.

**Keywords:** hydrogen ion concentration, saliva, salivary flow rate, toothbrushing, toothpastes

## Abstract

**Purpose:**

To determine the salivary flow rate and subsequent dilution of toothpaste and assess the pH of oral fluids during toothbrushing with toothpastes of various pHs.

**Materials and Methods:**

The study was conducted as an in-vivo trial involving 30 healthy volunteers. The participants took part in a series of trials distributed over four appointments. After a screening check, in which the participants’ stimulated and unstimulated salivary flow rate and buffering capacities were determined, four test series involving toothbrushing were conducted. Participants brushed their teeth using a manual toothbrush for 2 min: once without toothpaste and three times using toothpastes of varying pHs. The salivary flow rate and subsequent dilution of the toothpaste was determined. Additionally, the pH of the collected oral fluid was analysed.

**Results:**

Brushing teeth with toothpaste caused a statistically significant increase in salivary flow rate (median/IQR in ml/min) (Elmex Kariesschutz 3.29/1.36, Colgate Total Original 3.23/1.08, Elmex Sensitive Professional 3.18/1.39) when compared to brushing teeth using a manual toothbrush without toothpaste (1.85/0.78) (p < 0.05). The variation in pH of the oral fluid samples was dictated primarily by the pH of the toothpaste used.

**Conclusion:**

The salivary flow rate when brushing using toothpaste was similar across all tested toothpastes, independent of pH, and had an average median of 3.23 ml/min. The dilution of 1 g of toothpaste during a standard toothbrushing procedure of 2 min is therefore approximately at a ratio of one part toothpaste to 6.5 parts saliva.

Toothbrushing has become an integral part of our daily routine and remains the most widely implemented technique for home dental care. Brushing using fluoride toothpastes has proven to be one of the most important contributions to the reduction of dental disease and decay.^20,24^ As the prevalence of caries-related dental disease has seen an impressive decline in the last decades,^25^ other causes of dental hard tissue damage, such as erosion and abrasion, have become a new point of focus. Numerous studies investigate the effects of foods, drinks and dental materials such as toothpastes as sources of erosive and abrasive damage in in-vivo and in-vitro trials. Especially in-vitro methods have proven popular, as they offer the opportunity for more carefully controlled experimental settings. In terms of wear studies, examining the effects of toothbrushing on dental hard tissues through in-vitro models using human or xenogeneic specimens thus offers the advantage of being able to control many parameters. These include the brushing speed, force and frequency, as well as other factors such as temperature and amount of toothpaste and diluent applied. Such trials often involve the creation of toothpaste slurries, in which toothpaste is mixed with water or artificial saliva. The literature, however, does not provide a clear consensus concerning the optimum mixture that should be used.

In-vitro wear experiments typically employ methods which make use of fixed toothpaste concentrations. This is largely due to the fact that continually diluting the toothpaste throughout the act of brushing, as is the case in actual intraoral situations, is challenging. In the literature, concentrations of toothpaste to diluent (artificial saliva or water) are reported as varying from 1:1 to 1:5, with the most common being 1:3.^13,15,16,18,21,22,28,30–34,36^

To the authors’ knowledge, only a very limited number of in-vivo studies^9,19^ have examined the dilution of toothpaste during toothbrushing in healthy adults. This, however, is significant, as in-vitro studies should aim to replicate the in-vivo oral cavity environment as accurately as possible. Manly and Schickner^19^ aimed to determine the dilution of toothpaste throughout the toothbrushing process. They found a range of dilution from 0.2 to 3.9 parts of water and concluded that a dilution of 1:2 represented an accurate approximation to be used for in-vitro testing, as this fell in the middle of the range and was consistent with dilutions used in previous experimental settings. A further study on the topic was conducted by Duke and Forward,^9^ who studied conditions during brushing with toothpastes in an in-vivo study involving six subjects. They determined that, during an initial 30-s brushing phase, the slurry contained “22% paste and 78% saliva” (indicating a dilution of approximately 1:3.5), until the concentration was finally diluted down to below 10% of the initial concentration by the end of the toothbrushing procedure (lasting an average of 50 s, not including time spent expulsing the slurry and rinsing the toothbrush). Using the in-vivo data of both of the aforementioned studies, Franzò et al^14^ modelled the dilution of toothpaste during brushing. They concluded that the dilution factor of 1:2 was appropriate for use in in-vitro studies.

A further point of interest is the pH value of the intraoral fluid mixtures arising during toothbrushing in-vivo. As previously mentioned, toothpaste slurries are typically created on the basis of mixing toothpaste with either water or artificial saliva. While the main component of saliva is water, human saliva is a highly complex and variable secretory fluid with a sophisticated buffer system. A study by Zehnder et al^38^ demonstrated the relevance of such a buffer system for toothpaste abrasivity tests. In order to mimic intraoral situations in an in-vitro setting as accurately as possible, examination of the pH of intraoral fluids during toothbrushing in-vivo therefore seems relevant and necessary.

Numerous studies have examined the relationship between toothbrushing and salivary flow and composition. In addition to observing toothpaste concentrations during toothbrushing, Duke and Forward^9^ examined intraoral pHs during the first 30 s of toothbrushing. Using commercially available toothpastes of various pHs ranging from 4.2 to 9.6, they recorded the pH of the slurries produced by 5 subjects at 10 s and 30 s of the brushing process. They found that the variation in pH was primarily dependent on the toothpaste used and little affected by the variation in salivary content. The results indicated that both the acidic and alkaline toothpaste slurries became progressively more neutral from 10 s to 30 s as the buffering effect of the saliva was observed.

Given the scarcity of studies examining the dilution of toothpastes during toothbrushing in in-vivo studies, the present study aimed to provide insight into the dilution of toothpastes with various pHs during toothbrushing and hoped to provide a reference toothpaste dilution factor for future in-vitro studies. Furthermore, this study aimed to examine toothpaste slurry pHs during the toothbrushing process.

The null hypothesis formulated for the present study was that there is no difference in the salivary flow rate and subsequent dilution of toothpaste during toothbrushing with toothpastes of different pH values.

## Materials and Methods

### Ethics

This study was submitted to the local Cantonal Ethics Committee of Zürich (KEK-ZH 2021-01874) and registered with the German Clinical Trials Register (DRKS00026949). Approval was granted and the study was conducted in accordance with the authorised protocol and the Declaration of Helsinki, and followed the Good Clinical Practice (ICH-GCP) guidelines, as well as national legal and regulatory requirements. The study was classified as risk category A according to ordinance HRO Art.7.

### Project Population, Recruitment and Screening

The study aimed to include 30 healthy adult volunteers, 15 females and 15 males. The minimum age requirement was set at 18 years. To ensure an adequate number of participants, the study participants who dropped out of the study prematurely before data collection was completed were replaced by recruiting new participants. Thus, a total of 48 individuals (28 females and 20 males) were recruited from clinical staff, students, and daily clinical practice at the Center for Dental Medicine, University of Zürich, Switzerland. The volunteers were informed about the purpose and the procedures of the research project, as well as possible risks of the study, both in oral and written form. All test subjects confirmed their willingness to participate in the study by signing an informed consent form. Participants were additionally requested to fill out the patient information form to confirm their overall health status, including information on medication intake, drug use, allergies, pregnancy, etc.

Volunteers that failed to meet any of the inclusion criteria were not allowed to participate in the study. Exclusion criteria included:

- Salivation rates deviating from accepted norm values: unstimulated salivary flow > 0.4 ml/min, stimulated salivary flow > 2.0 ml/min
- Medication intake with known effects on salivary flow or intraoral pH
- Pregnancy
- Simultaneous participation in another clinical trial
- Known allergies or intolerances towards an ingredient in the test toothpastes
- Dental arches with less than 20 teeth
- Active orthodontic appliances at the time of the study (with the exception of a dental retainer wire)
- Open intraoral wounds
- Persons with dental appointments (dentist or dental hygienist) scheduled less than one day from study trial appointments
- Inability to give an informed consent or to follow protocol requirements and procedures

### Determination of Unstimulated and Stimulated Salivary Flow

During the preliminary screening check, participants’ stimulated and unstimulated salivary flow rates were determined. For the unstimulated salivary flow, the intraoral fluids were collected over a 15 min time-span. For the stimulated salivary flow rate, saliva produced while chewing on a paraffin sheet was collected over 5 min. The salivary samples were then weighed to determine the flow rate per minute, and the pH values and buffering capacities were measured using a laboratory titration device (848 Titrino plus, Metrohm; Herisau, Switzerland). Participants with salivation rates deviating from the pre-set norm values were excluded from the study. A total of 16 participants (4 males and 12 females) were excluded during the screening phase due to salivary flow rates below the limits set in the inclusion criteria. An overview of participant data including sex and salivary flow rate in ml/min for both stimulated and unstimulated salivary flow is presented in Table 1.

**Table 1:** Overview of participant data including sex and salivary flow rate in ml/min for both stimulated and unstimulated salivary flow

Subject	Sex (M/F)	Salivary flow rate (ml/min)	
		Unstimulated Salivary Flow / 15 min	Stimulated Salivary Flow / 5 min
1	M	0.81	3.40
2	M	0.44	2.02
3	M	1.96	3.68
4	M	0.66	2.48
5	M	1.09	3.36
6	M	0.92	2.05
7	M	0.63	2.34
8	M	1.16	2.97
9	M	1.08	3.02
10	M	0.83	2.32
11	M	0.76	2.08
12	M	1.17	2.79
13	M	1.68	2.38
14	M	1.04	2.63
15	M	0.64	3.68
1	F	1.05	2.67
2	F	0.91	3.85
3	F	1.44	3.25
4	F	1.67	2.81
5	F	0.60	2.38
6	F	0.76	2.70
7	F	0.78	2.27
8	F	0.96	2.69
9	F	1.24	3.17
10	F	0.83	3.71
11	F	0.46	2.75
12	F	0.82	3.87
13	F	1.07	2.23
14	F	0.60	3.95
15	F	1.49	3.41

### Toothbrushing Without Toothpaste

Participants were given a dry manual toothbrush (Paro M43, Esro; Kilchberg, Switzerland) and requested to brush their teeth for 2 min. Instruction on brushing techniques was not given. Participants were simply asked to brush their teeth according to their usual habits. Participants could choose to expel all the fluid at the end of the brushing period or intermittently, as seen fit. The oral fluid produced was collected in a test beaker. The toothbrush was weighed in its clean state prior to toothbrushing and then once more while covered with remains of oral fluid, after the toothbrushing was complete. The weight of the clean toothbrush was then subtracted from the weight of the toothbrush covered in fluid to determine the weight of the oral fluid remaining on the toothbrush. The weight of the oral fluid collected in the test beaker as well as the weight of the oral fluid remaining on the toothbrush were summed to determine the total weight of oral fluid produced. From this, the toothbrushing salivary flow rate per minute for toothbrushing without toothpaste was calculated and the pH of the fluid sample was determined.

### Toothbrushing with Three Toothpastes of Varying pH

At three appointments, scheduled on separate days, participants were instructed to brush their teeth for 2 min using a dry manual toothbrush coated with 1 g of toothpaste (all by Colgate-Palmolive; Świdnica, Poland) of varying pH and chemical composition (A: Elmex Kariesschutz pH 4.7; B: Colgate Total Original pH 6.9; C: Elmex Sensitive Professional pH 9.0). The pH values of the toothpastes were taken as determined by Tawakoli et al.^29^ The selection of three toothpastes was made based on the following criteria: the toothpastes were approved for human application, were commercially available to consumers in local supermarkets, and had differing pH values, ranging from slightly acidic to neutral and alkaline. The further chemical composition of the toothpastes was not taken into consideration in the selection process. The chemical composition of the toothpastes used is presented in Table 2.

**Table 2:** Detailed chemical composition of the toothpastes used according to the manufacturer

Tested toothpastes (Manufacturer)	pH	Composition
Elmex Kariesschutz (Colgate-Palmolive, Świdnica, Poland)	4.7	Aqua, hydrated silica, sorbitol, hydroxyethylcellulose, saccharin, limonene, CI 77891, Aroma (> 100 PPM): anethole, carvone, eucalyptol, limonene, *Mentha arvensis* extract, *Mentha piperita* (peppermint) oil, *Mentha viridis* (spearmint) leaf oil, menthol. Stannous fluoride, olaflur (amine fluoride); total fluoride content: 1400 ppm F^-^
Colgate Total Original (Colgate-Palmolive)	6.9	Glycerin, aqua, hydrated silica, sodium lauryl sulfate, arginine, aroma (> 100 PPM): anethole, benzyl alcohol, (e)-3-benzo[1-3]dioxol-5-yl-N-N-diphenyl-2-propenamide, *Illicium verum* (anis) fruit/seed oil, *Mentha arvensis* extract, *Mentha piperita* (peppermint) oil, *Mentha viridis* (spearmint) leaf oil, menthol, menthone. Cellulose gum, zinc oxide, poloxamer 407, zinc citrate, tetrasodium pyrophosphate, xanthan gum, benzyl alcohol, cocamidopropyl betanine, sodium fluoride (1450 ppm F^-^), sodium saccharin, phosphoric acid, sucralose, CI 77891
Elmex Sensitive Professional (Colgate-Palmolive)	9.0	Arginin 8%, calcium cardonate, aqua, sorbitol, aroma (> 100 PPM): anethole, carbonic acid 2-hydroxyethyl 5-methyl-2-(1-methylethyl)cyclohexyl ester, Carbonic acid 2-hydroxypropyl 5-methyl-2-(1-methylethyl)cyclohexyl ester, ethyl menthane carboxamide, eucalyptol, isomenthone, limonene, *Mentha arvensis* extract, *Mentha piperita* (peppermint) oil, menthol, thymol. Poloxamer 407, sodium monofluorophosphate (1450 ppm F^-^), cocamidopropyl betaine, zinc oxide, benzyl alcohol, cellulose gum, zinc citrate, sodium bicarbonate, tetrasodium pyrophosphate, xanthan gum, sodium saccharin, sucralose, limonene, CI 77891.

pH data according to Tawakoli et al.^29^

The fluid (saliva/toothpaste mixture) produced was collected in a test beaker. The weight of the collected fluid mixture and the weight of the fluid mixture remaining on the toothbrush were summed using the same method as described above. One gram was then subtracted to compensate for the weight of the toothpaste, and thus the total weight of actual oral fluid produced was determined. Following this, the toothbrushing salivary flow rate per minute for toothbrushing with toothpaste was calculated; the pH of the fluid sample was also determined.

One participant withdrew from the study after the 3rd appointment and a further participant was excluded from the trial after a sample was determined unusable due to contamination with blood from an intraoral wound. The results from study participants who did not complete all the trial tests were analysed, but not included in the final evaluation.

**Fig 1:**
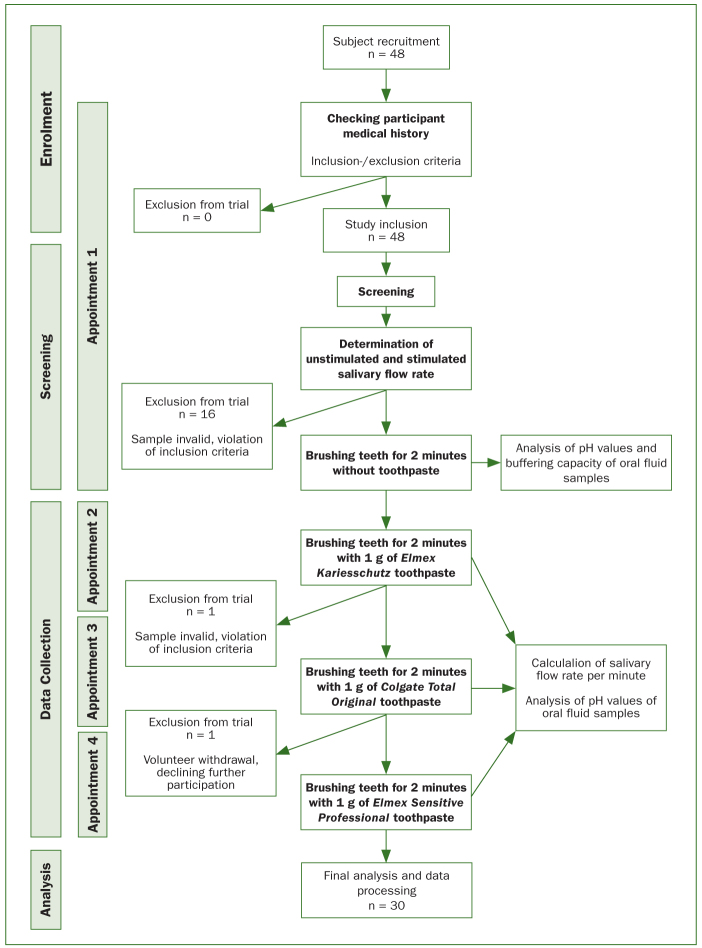
Fluxogram: schematic overview of subject recruitment and intervention with all study appointments.

A schematic overview of subject recruitment and intervention with all study appointments is presented in the Fluxogram in Fig 1.

### Statistical Analysis

Statistical analysis was conducted using the statistical software R (R Foundation for Statistical Computing, Vienna, Austria, URL https://www.R-project.org/.), including the Tidyverse 35 package. As the present study followed a within-subject repeated-measures design, a pairwise Wilcoxon signed-rank Test was conducted. The significance level was set at p ≤ 0.05. The resulting p-values were adjusted for multiple comparison using the Holm method.

## Results

**Fig 2:**
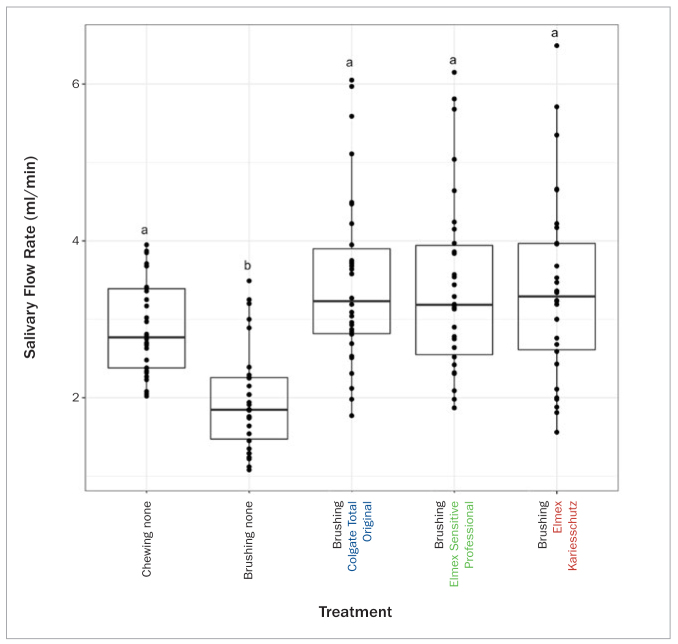
Boxplot of salivary flow rate (ml/min) displaying individual data points as well as median values and upper and lower quartiles. Chewing none: Stimulated salivary flow chewing on paraffin. Brushing none: 2 min toothbrushing without toothpaste. Brushing Colgate Total Original: 2 min brushing with Colgate Total Original. Brushing Elmex Sensitive Professional: 2 min brushing with Elmex Sensitive Professional. Brushing Elmex Kariesschutz: 2 min brushing with Elmex Kariesschutz. Statistical similarity is indicated by identical letters (a/b).

The salivary flow rates while chewing and brushing with and without toothpastes are presented in Fig 2 and Table 3.

**Table 3:** Salivary flow rate (ml/min) with mean ($\bar{x}$), standard deviation (SD σ), median, interquartile-range (IQR), minimum (min) and maximum (max)

Salivary flow rate (ml/min)						
Treatment	Mean ($\bar{x}$)	SD (σ)	Median	IQR	Min	Max
Chewing none	2.90	0.60	2.77	1.01	2.02	3.95
Brushing none	1.97	0.66	1.85	0.78	1.08	3.49
Brushing Colgate Total Original	3.50	1.12	3.23	1.08	1.77	6.05
Brushing Elmex Sensitive Professional	3.43	1.15	3.18	1.39	1.87	6.15
Brushing Elmex Kariesschutz	3.38	1.18	3.29	1.36	1.56	6.49

Brushing teeth using toothpaste caused a statistically significant increase in salivary flow rate (median/IQR in ml/min) (Elmex Kariesschutz 3.29/1.36, Colgate Total Original 3.23/1.08, Elmex Sensitive Professional 3.18/1.39) when compared to brushing teeth using a manual toothbrush without toothpaste (1.85/0.78) (p < 0.05, respectively). Salivary flow stimulated by a chewing motion (stimulated salivary flow rate) was not statistically significantly different (2.77/1.01) from the salivary flow achieved when brushing the teeth using toothpastes. Toothbrushing without toothpaste showed a statistically significantly lower salivary flow rate when compared to the salivary flow rate with stimulation by chewing.

**Fig 3:**
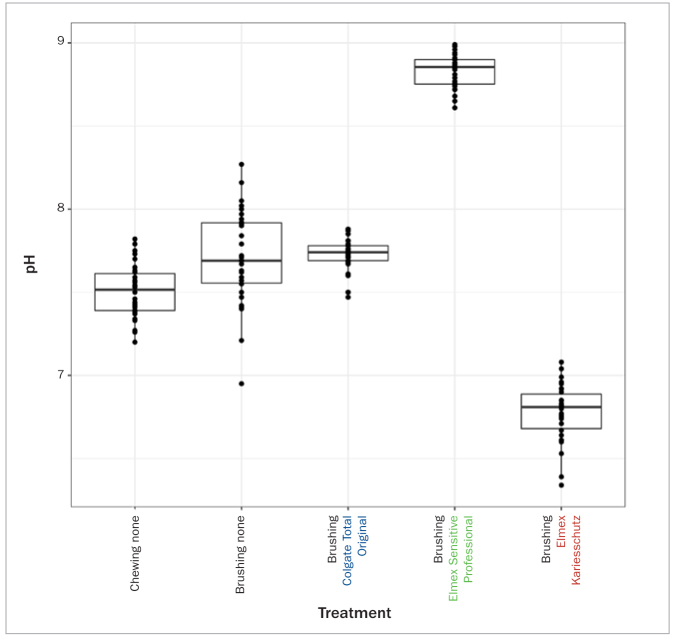
Boxplot of pH values of oral fluids displaying individual data points as well as median values and upper and lower quartiles. Chewing none: Stimulated salivary flow chewing on paraffin. Brushing none: 2 min toothbrushing without toothpaste. Brushing Colgate Total Original: 2 min brushing with Colgate Total Original, pH 6.9. Brushing Elmex Sensitive Professional: 2 min brushing with Elmex Sensitive Professional, pH 9.0. Brushing Elmex Kariesschutz: 2 min brushing with Elmex Kariesschutz, pH 4.7.

The pH values of oral fluids while chewing and brushing with and without toothpastes are presented in Fig 3 and Table 4.

**Table 4:** pH values of oral fluids with mean mean ($\bar{x}$) , standard deviation (SD σ), median, interquartile-range (IQR), minimum (min) and maximum (max).

pH value						
Treatment	Mean ($\bar{x}$)	SD (σ)	Median	IQR	Min	Max
Chewing none	7.70	0.16	7.52	0.22	7.20	7.82
Brushing none	7.71	0.28	7.69	0.36	6.95	8.27
Brushing Colgate Total Original	7.72	0.10	7.74	0.09	7.47	7.88
Brushing Elmex Sensitive Professional	8.83	0.10	8.86	0.15	8.61	8.99
Brushing Elmex Kariesschutz	6.78	0.17	6.81	0.21	6.34	7.08

The analysis of the pH of the oral fluid samples was limited to descriptive statistics. Brushing using Elmex Kariesschutz, as a more acidic toothpaste, and Elmex Sensitive Professional, as a more alkaline toothpaste, resulted in oral fluids (median/IQR) of a more acidic (pH 6.81/0.21) and alkaline (pH 8.86/0.15) nature, respectively. More neutral pHs were observed for oral fluid collected throughout chewing (pH 7.52/0.22), brushing without toothpaste (pH 7.69/0.36) and brushing using Colgate Total Original (pH 7.74/0.09).

## Discussion

This study aimed to assess the salivary flow rate and subsequent dilution of toothpaste, as well as the pHs of oral fluids during toothbrushing with toothpastes of various pHs in an in-vivo setting. With the high prevalence of in-vitro studies using toothpaste slurries, such human trials are of great consequence for in-vitro studies, as they strive to mimic the in-vivo intraoral situation as accurately as possible.

### Test Subject Recruitment and Participation

Affoo et al^3^ studied whole salivary flow rates during toothbrushing in 21 volunteers aged 60 and older, and Ligtenberg et al^17^ recruited 80 healthy volunteers distributed into four test groups with 20-30 persons per group, to study changes in salivary secretion rate, buffering capacity and pH during toothbrushing. Duke and Forward^9^ and Manly and Schickner^19^ had only 6 subjects each. Due to their different designs, these studies could not be used for a preliminary power calculation. Since no other data were available for the present study design, it was difficult to estimate the appropriate number of participants. The aim was to include a total of 30 participants (15 females and 15 males, at least 18 years of age) in the study.

The present study is a foundational research project and is classified as a risk category A according to ordinance HRO Art.7. The study procedure itself was associated with no or very little risk for the project participants, as the methods used for data collection are all standardised, established, minimally invasive and not associated with any pain or discomfort. Potential risks included an intolerance or allergy towards the tested toothpastes or the paraffin used in determining the stimulated salivary flow rate. This however, was highly unlikely, as the toothpastes selected for the trial are approved for human application and are commercially available in local supermarkets. If participants were known to have sensitivities towards any ingredients or components of the tested toothpastes they were excluded from the trial. Determining stimulated salivary flow using a paraffin paper as a masticatory aid is standard procedure in dentistry and not associated with any great risks. The participants involved were all adults and capable of responsible, conscious chewing, so potential choking risks were minimised. Participants were given a new manual toothbrush with every trial test, which was replaced after a single use, so as to ensure there was no salivary contamination.

### Methods and Materials of Investigation

The methods chosen were appropriate to address the objectives of this investigation. A literature search was conducted to help determine details of the method, such as brushing time and amount of toothpaste to be used, to ensure that the in-vivo trial emulated real-life toothbrushing as closely as possible.

Very little is known about the actual amount of toothpaste that should be used; common phrases such as “a pea sized amount”^2,12^ never precisely quantitatively define the amount. The values found for the amount of fluoride toothpaste used with manual toothbrushes in other studies varies from 0.74 g ± 0.38 g (ongoing study at the Center for Dental Medicine) to 1.45 g ± 0.31 g.^9^ Further studies^4,27^ report values of approximately 1 g to 1.5 g. Therefore, it was decided that a total of 1 gram of toothpaste would be used, as this corresponds to the mean amount of toothpaste used in other studies.

A general consensus exists among oral healthcare professionals and national associations worldwide,^1,2,8,10,12,23^ recommending a brushing time of at least 2 min twice a day. Various studies have shown that actual brushing times differ, sometimes greatly, from this recommendation.^4,5,7,27,37^ Studies observing toothbrushing in adults report brushing times in a range of under 1 min to over 2.5 min.^4,11,26,27,37^ Thus, a total brushing time of 2 min was chosen, as this represents a good compromise of the previously observed real brushing times^4,6,11,26,27,37^ and the recommendations by oral health care professionals and national associations worldwide.

While the studies by Manly and Schickner^19^ and Duke and Forward^9^ were similar to the present study in terms of purpose, the current authors felt that the methodologies of those studies made their results too difficult to apply to the present day. Subjects in the studies by Manly and Schickner (1944)^19^ and Duke and Forward (1982)^9^ brushed their teeth for only 60 s and 50 s, respectively, which does not correspond to the currently favoured brushing time of 2 min. In addition, these studies were carried out in the 1940s and 1980s, respectively, and while Manly and Schickner^19^ experimented only with 1 toothpaste and Duke and Forward^9^ used 7 toothpastes of pH range similar to that used in our study, the current authors felt it would be appropriate to gather new data with toothpastes common today, which almost certainly differ in composition to those used so many years ago. The three toothpastes used in the current study were selected from commercially available brands to cover a range of pH values from slightly acidic, to neutral and alkaline. A limitation of the current study design is that the details of the chemical composition of the toothpastes were not taken into further consideration. However, as the results showed no statistically significant difference in flow rate across the three toothpastes tested, it seems that the differences in chemical composition are negligible in terms of effect on salivary flow rate. This, however, may be an interesting point for further investigation, and the study design might be expanded to include other commonly used toothpastes, taking the details of chemical composition into consideration, possibly increasing the generalisability of the results.

Due to the given differences (taste, smell etc.) of the toothpastes used and the control (toothbrushing without toothpaste), blinding was not possible for either the investigator or the study participants. However, following data collection, prior to any statistical analysis, the patient data was anonymised so that the evaluation of the data and results occurred in a blinded fashion.

### Discussion of Results

#### Salivary flow rate and subsequent toothpaste dilution

The null hypothesis of the present study was that there is no difference in the salivary flow rate and subsequent dilution of toothpaste during toothbrushing with toothpastes of different pH values. This null hypothesis cannot be rejected, as no statistically significant difference in the salivary flow rate was observed when brushing with the investigated toothpastes.

The salivary flow stimulated by a chewing motion (median/IQR 2.77/1.01 ml/min) was not statistically significantly different from the stimulated salivary flow achieved when brushing teeth using toothpastes. However, toothbrushing without toothpaste showed a statistically significantly lower salivary flow rate when compared to the salivary flow rate through stimulation by a chewing motion. This would indicate a smaller contribution of the toothbrush itself in providing mechanical stimulation for salivation when compared to stimulation by masticatory (chewing) movement. Brushing teeth using toothpaste caused a statistically significant increase in salivary flow rate (median/IQR in ml/min) (Elmex Kariesschutz 3.29/1.36, Colgate Total Original 3.23/1.08, Elmex Sensitive Professional 3.18/1.39) when compared to brushing teeth using a manual toothbrush without toothpaste (1.85/0.78) (p < 0.05, respectively). This is consistent with the findings of Ligtenberg et al.^17^ Thus, it seems that the increase in salivary flow rate may be primarily attributed to the gustatory stimulation via the toothpaste. An interesting point of further investigation would be to conduct a similar study using not only manual, but also electric or sonic toothbrushes. In addition, it might prove beneficial to instruct participants on a standardised appropriate brushing technique, thus overcoming a further limitation in the current study design.

The salivary flow rate when brushing using toothpaste was similar across all tested toothpastes, independent of their pH. The average median salivary flow rate during toothbrushing with toothpaste was 3.23 ml/min, showing a dilution of 1 g of toothpaste during a standard toothbrushing procedure of 2 min to be 1:6.46. This, of course, is only a momentary view of the actual physiological situation as it occurs in the tooth-brusher’s mouth. In reality, there is a continual dilution of the toothpaste throughout the act of brushing and thus, the longer the toothbrushing procedure is carried out, the more dilution occurs. In addition, the actual concentration of the toothpaste in the intraoral slurry may further vary, as individuals do not necessarily retain the entire mixture in their mouths throughout the whole toothbrushing process, expulsing excesses of slurry intermittently, resulting in an even higher dilution of the toothpaste. This implies further variations in toothpaste concentration. Duke and Forward^9^ studied the conditions occurring while brushing with toothpastes in an in-vivo study. They observed that subjects brushed their teeth for 50 s on average, cleaning the brush with water and expulsing the slurry intermittently throughout 4 brushing phases. They found that after the initial 30 s brushing phase, almost 60% of the toothpaste had been expelled. Albertsson et al^4^ reported that 60% of participants spat out part of the toothpaste-saliva slurry during brushing, while 15% retained the mixture and spat after brushing. Such behavioural variation is not something which was controlled or monitored in this study. Participants could choose to expel all the fluid at the end of the brushing period or intermittently as seen fit. It should also be noted that there was likely a discrepancy between the toothbrushing performance as it occurred in the laboratory setting in this study and the actual toothbrushing performance by individuals at home without supervision. While the actual time spent by individuals brushing their teeth also varies greatly, the international recommendations are set at 2 min brushing time, and thus a measurement of the dilution within this timeframe seemed appropriate.

This study found values for the dilution of the toothpaste concentration which differ in comparison to those found in other in-vivo studies. Manly and Schickner^19^ found a range of dilution of toothpaste throughout the toothbrushing process. Subjects were given a manual brush and requested to brush their teeth as they normally do. They reported, that some subjects wet the brush with water before applying the toothpaste and that this caused a dilution of toothpaste by a factor of 0.2 parts. Such an additional dilution was not taken into consideration in this study as the toothpaste was applied directly to the dry toothbrush and not wet, prior to use by the participants. Manly and Schickner^19^ then measured the dilution of the toothpaste after 30 s and 60 s and found it to have increased to 1.4 and 3.9 parts, respectively. They concluded that a dilution of 1:2 represents an accurate approximation, as this value lies in the middle of the range. Because the present study measured only the complete dilution as it occurs after 2 min, a direct comparison is not possible. However, since the dilution of the toothpaste in-vivo continually increases with time, the higher dilution of approximately 1:6.5 achieved after 2 min in this study seems plausible.

In comparison, Duke and Forward^9^ determined that during an initial 30-s brushing phase, the slurry contained “22% paste and 78% saliva”, indicating a dilution of approximately 1:3.5. They reported that the concentration had been diluted down to <10% (indicating a dilution of approximately 1:10) of the initial concentration by the end of toothbrushing after 50 s. However, as participants in the aforementioned study expelled almost 60% of the initial toothpaste and cleaned the brush with water after 30 s and repeated this once again after brushing for an additional 10 s and twice more after an additional 5 s, a comparison of the results beyond the 30-s interval is not appropriate. In addition, it should be mentioned that, unlike the present study and Manly and Schickner’s,^19^ Duke and Forward^9^ did not use weight ratios to estimate toothpaste concentrations. Instead, they measured the fluoride content contained in the slurries and compared it to the fluoride level found in the toothpaste, thus estimating the amount of toothpaste present in each slurry. A comparison of the dilution observed at 30 s (1:3.5)^9^ vs the dilution of approximately 1:6.5 observed in this study after 2 min vs the dilution of 1:1.4 and 1:3.9 parts after 30 s and 60 s,^19^ respectively, suggests a large discrepancy between in-vivo results. This tendency can be observed within the current study population as well. The salivary flow rate while toothbrushing with toothpastes varied from a minimum of ca. 1.7 ml/min (Colgate 1.77 ml/min, Elmexgreen 1.87 ml/min, Elmexred 1.56 ml/min) to a maximum of ca. 6.2 ml/min (Colgate 6.05 ml/min, Elmexgreen 6.15 ml/min, Elmexred 6.49 ml/min). This represents a more than 3.5-fold increase and is indicative of the large range in physiological salivary flow rate. These values may be attributed to individuals who consistently had a high or low salivary flow rate across all toothpastes.

While this study found a dilution of toothpaste at a ratio of nearly 1:6.5 after 2 min, previous in-vitro studies have typically used more concentrated slurries; the most commonly used dilution factor was 1:3. Assuming that the average individual follows the international recommendations and brushes their teeth for 2 min, a median flow rate of approximately 3.2 ml/min and therefore a dilution of the toothpaste at a ratio 1:3.2 after 1 min seems a reasonable approximation. Therefore, the present authors believe a dilution of 1:3 to be a realistic average representation of in-vivo conditions which can used in in-vitro trials with brushing periods of 1 min. If a longer brushing period of 2 min is performed, a higher dilution of 1:6.5, as observed in the present study, should be applied. It should be noted that it is unclear whether the dilution rate per minute may be applied proportionally for brushing periods lasting longer than 2 min.

#### pH values

The analysis of the pH of the oral fluid samples showed similar, more neutral pHs for oral fluid collected throughout chewing, brushing without toothpaste and brushing using Colgate Total Original, a more neutral toothpaste with a pH of 6.9. Brushing with Elmex Kariesschutz, a more acidic toothpaste with a pH of 4.7 and Elmex Sensitive Professional, a more alkaline toothpaste with a pH of 9.0, resulted in more acidic and more alkaline oral fluids, respectively. This is consistent with the findings of Duke and Forward.^9^ They found that the variation in pH of the slurries collected at 10 s and 30 s of brushing was influenced only very slightly by differences in salivary content, and instead could be primarily attributed to the toothpaste being used. Those authors experimented with commercially available toothpastes of various pHs, ranging from 4.2 to 9.6, and found that both the acidic and alkaline toothpaste slurries became progressively more neutral from 10 s to 30 s as the buffering effect of the saliva took effect. This raises an interesting aspect for further investigation, as this study recorded only a single measurement at the end of the toothbrushing act. It might prove interesting to observe how the pH varies as toothbrushing progresses.

## Conclusion

The salivary flow rate when brushing teeth using a manual toothbrush is similar across all tested toothpastes, independent of their pH. The average median salivary flow rate observed during toothbrushing with toothpaste was 3.23 ml/min, revealing a dilution of 1 g of toothpaste during a standard toothbrushing procedure of 2 min to be nearly 1:6.5. Therefore, a dilution of the toothpastes after 1 min occurs at a ratio of approximately 1:3, which might represent an appropriate dilution factor to be used in an experimental setting with 1-min brushing periods. If longer brushing periods of 2 min are performed, a higher dilution of 1:6.5 should be used.
